# Royal Jelly Facilitates Restoration of the Cognitive Ability in Trimethyltin-Intoxicated Mice

**DOI:** 10.1093/ecam/nep029

**Published:** 2010-10-25

**Authors:** Noriko Hattori, Shozo Ohta, Takashi Sakamoto, Satoshi Mishima, Shoei Furukawa

**Affiliations:** ^1^Nagaragawa Research Center, API Co., Ltd, Nagara, Gifu 502-0071, Japan; ^2^Laboratory of Molecular Biology, Gifu Pharmaceutical University, Daigaku-nishi 1-25-4, Gifu 501-1196, Japan

## Abstract

Trimethyltin (TMT) is a toxic organotin compound that induces acute neuronal death selectively in the hippocampal dentate gyrus (DG) followed by cognition impairment; however the TMT-injured hippocampal DG itself is reported to regenerate the neuronal cell layer through rapid enhancement of neurogenesis. Neural stem/progenitor cells (NS/NPCs) are present in the adult hippocampal DG, and generate neurons that can function for the cognition ability. Therefore, we investigated whether royal jelly (RJ) stimulates the regenerating processes of the TMT-injured hippocampal DG, and found that orally administered RJ significantly increased the number of DG granule cells and simultaneously improved the cognitive impairment. Furthermore, we have already shown that RJ facilitates neurogenesis of cultured NS/NPCs. These present results, taken together with previous observations, suggest that the orally administered RJ may be a promising avenue for ameliorating neuronal function by regenerating hippocampal granule cells that function in the cognition process.

Royal jelly (RJ) is a substance synthesized by the lower pharyngeal gland of the honeybee, and is thought to play important nutritional roles for the queen honeybee. RJ consists mainly of proteins, sugars, lipids, vitamins and free amino acids [1–3], and has a variety of biological activities toward various types of cells [4–7]. Earlier we reported that RJ induces neurite outgrowth from cultured PC12 cells [8, 9], a cell line of rat pheochromocytoma, via adenosine A_2A_ receptors, and enhances the phosphorylation of both extracellular signal-regulated kinase 1 or 2 (ERK1/2) and cAMP-response element-binding protein (CREB) in both PC12 cells [9, 10] and cultured neural stem/progenitor cells (NS/NPCs) [11]. cAMP-dependent signaling plays crucial roles in the hippocampal long-term potentiation associated with the ability for learning and memory [12, 13]. These observations suggest that RJ can activate intracellular signaling pathways by acting through adenosine A_2A_ receptors, with the result being hippocampal long-term potentiation. However, there are no reports so far showing the effects of RJ on the recognition ability for learning and memory.

Trimethyltin (TMT) is a toxic organotin compound that induces acute neuronal death selectively in the hippocampal dentate gyrus (DG) [14–16] followed by cognition impairment [17, 18]. However, the TMT-injured hippocampal DG itself is reported to regenerate the neuronal cell layer through rapid enhancement of neurogenesis [16]. NS/NPCs are present in the adult hippocampal DG, and they can generate neurons that can function in cognition. Furthermore, we have already shown that RJ facilitates neurogenesis of cultured NS/NPCs. Therefore, TMT-treated mice are a useful animal model to study neurogenesis in association with the ability for learning and memory.

In this study, we examined whether RJ could ameliorate TMT-induced cognitive impairment.

## 1. Materials and Methods

### 1.1. Materials

TMT was purchased from Sigma (St Louis, MO); and toluidine blue, from WALDECK-Gmbh & CoKG (Munster, Germany). RJ produced by *Apis melifera* (lot no T07080728R) was provided by Api Co., Ltd (Gifu, Japan). Chemical composition analysis demonstrated that the RJ used for the present study consisted of moisture (63.6%), proteins (14.5%) and 10-hydroxy-trans-2-decenoic acid (1.98%), demonstrating that a quite usual RJ was used. The RJ was lyophilized to a powder and mixed with powdered regular food (1% or 5% w/w).

### 1.2. Animals and Treatment of Animals

Male ICR mice (7 weeks old, 33–40 g body weight) were purchased from Japan SLC, Inc. (Shizuoka, Japan), and maintained in a controlled environment (23 ± 1°C, 55 ± 10% humidity) with a 12 h light–dark (8:00–20:00) cycle, and allowed unlimited access to food and water. The animals were cared for according to the international guidelines set out in the NIH publication *Principles of Laboratory Animal Care*.

The mice were divided into two groups (*n* = 24 each). One group was treated with a single injection of TMT (2 mg kg^−1^, TMT group); and the other, with an injection of phosphate-buffered saline [PBS], PBS group). Each group was subdivided into three groups fed regular food (control, *n* = 8 or 10) or regular food containing 1% or 5% RJ (*n* = 8, each) 2 days after the treatment with TMT or vehicle. There was no difference among the three groups in behavioral scores at the time of subdivision. After 6 days of consumption of the various diets the mice were subjected to behavioral tests.

### 1.3. Spontaneous Alternation Test in a Y-Maze

The maze was made of brown water-repellent corrugated cardboard; each arm was 40 cm long, 12 cm high, 3 cm wide at the bottom and 10 cm wide at the top. The arms converged at an equilateral triangular central area that was 4 cm at its longest axis. The procedure was basically the same as described previously [19]. Each mouse was placed at one of the arms of the apparatus and allowed to move freely through the maze during an 8 min session. The series of arm entries was recorded. Alternation was defined as successive entry into the three arms on overlapping triplet sets. Alternation behavior (%) was calculated as the ratio of actual alternations to possible alternations (defined as the number of arm entries minus 2) multiplied by 100.

All results were expressed as the mean ± SE for each group. Simple comparisons of the means and SE of data were performed by using Student's *t*-test. Multiple comparisons between groups were made by one-way ANOVA, and then differences among means were analyzed by performing Tukey's test for the same number of experimental samples in a group or Holm's test for different ones in a group.

### 1.4. Biochemical Analyses

After the behavioral test battery, the mice were sacrificed by asphyxiation, and their brains were removed and cut in half sagittally. The hemispheres were randomly chosen from each group for each biochemical analysis, and embedded in paraffin. Serial brain sections of 8 *μ*m in thickness including the hippocampal DG were prepared, from which five sections were selected, with one of every two consecutive sections taken. The sections were stained with neuron-specific Nissl staining using 0.5% toluidine blue solution for 25 min. They were then dehydrated, incubated in ammonia solution for 5 min, and then enclosed in resin that had been dissolved in xylene. The number of the neurons with a diameter of over 10 *μ*m was counted without any biases in 1 mm^2^ areas of the DG granule cell layer.

## 2. Results

### 2.1. TMT Reduced Neuronal Cell Number and Impaired Alteration Behavior Rate

**Figure 1:**
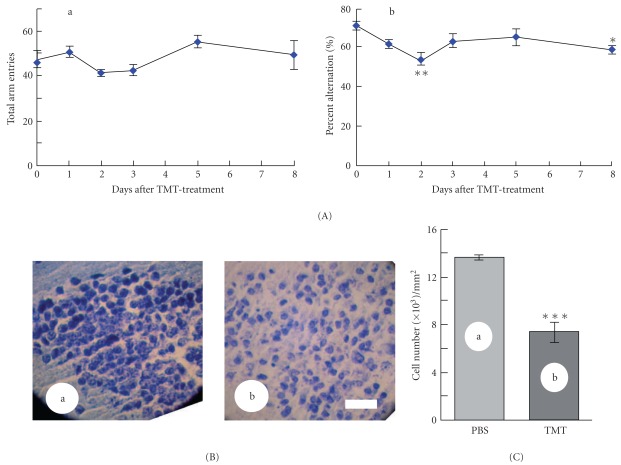
Effect of TMT on the spontaneous alternation behavior (A) and number of neurons in the hippocampal DG (B and C). (A) Mice were injected with a single dose of TMT (2 mg kg^−1^), and subjected to the spontaneous alternation test in the Y-maze every day for 8 days after the day of TMT injection. The ordinate shows the total alternation opportunities (total arm entries, a) and percentage alternation (b) for 8 min in each test. The values (the mean ± SE; *n* = 3–5) obtained 2 or 8 days after the treatment were significantly lower than the original value, determined by one-way ANOVA with Tukey's test, *P <  .05 and **P <  .01, respectively. (B) Photographs of Nissl-stained hippocampal DG of mice 2 days after treatment with PBS (a), or TMT (b). Scale bar: 50 *μ*m. (C) Number of granule cells in the hippocampal DG 2 days after PBS (a) or TMT (b) treatment. The values are expressed as the mean ± SE (*n* = 4). The difference between the two values was determined by Student's *t*-test to be significant at ***P <  .001.

To evaluate the effect of TMT on short-term memory or neuronal damage of the DG, we intraperitoneally injected a single dose of TMT into mice (2.0 mg kg^−1^) at day 0. TMT attenuated the spontaneous alternation behavior first on day 2, which was restored on days 3 or 5, but impaired it secondarily on day 8 (Figure 1(A)). As there was no significant difference in the number of arm entries (data not shown), mice used were suggested to have the same levels of motivation, curiosity and motor function. Namely, TMT caused both acute (day 2) and non-acute (day 8) impairments of the alternation behavior.

To clarify a link of the alternation behavioral drawback to the neuronal damage of the DG, we focused on the acute effect that occurred on experimental day 2. The brain sections from mice on day 2 were Nissl-stained, and evaluated for neuronal damage to the DG. The TMT-treated mice showed nearly a 50% marked reduction in the number of DG neurons (Figures 1(B) and 1(C)), suggesting that a half of the DG neurons were vulnerable to acute toxicity of TMT and that this neuronal loss was responsible for the impaired acute spontaneous alternation behavior (Figure 1(A)). These observations are in good agreement with those of a previous report [16], which suggested that neurogenesis in the DG occurs several days after TMT treatment. Therefore, the behavioral restoration found on experimental days 3 or 5 (Figure 1(A)) is likely to be a consequence of the neurogenesis spontaneously evoked in the DG. In other words, TMT might acutely induce neuronal death in the DG on day 2, and later attenuate neurogenesis in the DG around day 8. Therefore, we next evaluated the ameliorating effect of RJ on the alternation behavior and the cell number of the DG on day 8.

### 2.2. RJ Attenuated TMT-Induced Impairment of Spontaneous Alternation Rate and Neuronal Damage

**Figure 2:**
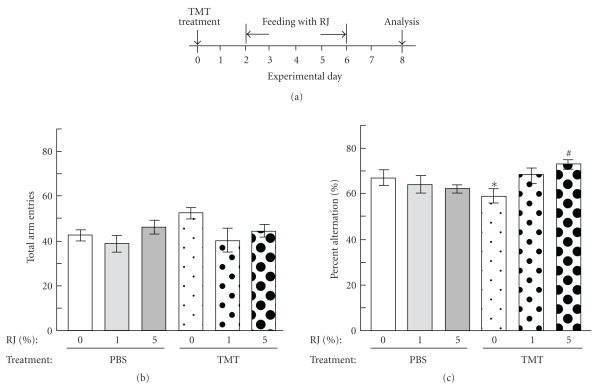
Amelioration of the TMT-induced impairment of short-term memory by the feeding with RJ. Mice were processed according to the schedule illustrated in (a). The mice were treated with TMT in PBS or PBS alone on day 0, and feeding with RJ (0, 1 or 5%) for 6 days was started from day 2. The spontaneous alternation behavior test was performed on day 8. The ordinates in (b) and (c) show the total alternation opportunities (total arm entries) and percentage of alternation, respectively, measured for 8 min. The values are expressed as the mean ± SE (*n* = 8–10). The difference between the control value (PBS-treated mice fed without RJ) and that for the TMT-treated mice fed without RJ diet was significant at *P <  .05 by one-way ANOVA with Holm's test. ^#^ *P* <  .05, TMT-treated mice with versus without RJ (5%).

**Figure 3:**
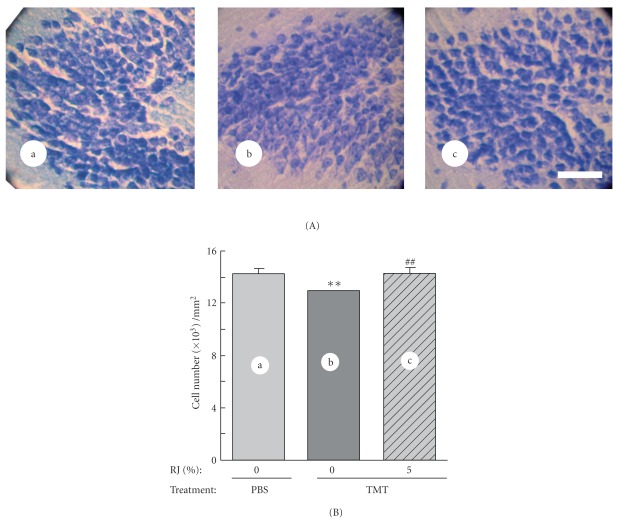
Amelioration by RJ of the TMT-induced damage to the hippocampal DG. (A) Photographs of Nissl-stained hippocampal DG of mice 8 days after PBS (a), or TMT (b) treatment, or TMT treatment + 6-day feeding of 5% RJ diet (c). Scale bar: 50 *μ*m. (B) Number of granule cells in the hippocampal DG of mice 8 days after PBS (a), or TMT treatment (b), or TMT treatment + 6-day feeding of 5% RJ diet (c). The values are expressed as the mean ± SE (*n* = 4). The difference between the control value (PBS-treated mice fed without RJ) and that for TMT-treated mice fed without RJ diet was significant at **P <  .01 by Tukey's test. ^##^ *P* <  .01, TMT-treated mice with versus without RJ.

According to the experimental schedule shown in Figure 2(a), mice were injected with TMT in PBS or PBS alone on day 0 and successively fed the RJ-containing diet for 6 days, from days 2 to 7; and the spontaneous alternation rate and neuronal cell number were then evaluated on day 8. TMT treatment significantly impaired the spontaneous alternation rate when evaluated on day 8; however, the feeding with the diet of 5% RJ, but not 1% RJ, significantly suppressed this impairment (Figure 2(c)). As there was no significant difference in the number of arm entries among the groups (Figure 2(b)), RJ did not affect the locomotion activity. After the behavioral test, the mice were fixed with 4% paraformaldehyde solution, and brain sections were prepared and stained. As shown in Figure 3(A), TMT-treated mice without the RJ diet (A, b) appeared to have a smaller number of neurons than TMT-non-treated mice without the RJ diet (A, a); whereas the TMT-treated mice with the RJ diet (A, c) appeared to have a greater number of neurons than TMT-treated ones without it. Actual cell counting demonstrated that the TMT-induced decrease in the number of neurons on day 8 (Figure 3(B), b) was significantly attenuated by RJ feeding (Figure 3(B), c).

## 3. Discussion

It is known that NS/NPCs reside in the hippocampal DG of adult animals, where these cells proliferate and differentiate into neurons [20–23] in response to injury [21, 22], suggesting that the activation of NS/NPCs by some agents or substances to promote neurogenesis facilitates repair of the injured brain. Neurogenesis in the adult DG is largely responsible for regulating the number of granule cells, which cells are involved in the cognitive ability [24].

In this study, we aimed at evaluating if orally administered RJ could facilitate the generation of hippocampal DG granule cells *invivo*. TMT is reported to induce acute neuronal death selectively in the DG [14–16], but the neurons substantially regenerate in several days, suggesting that TMT-treated mice are useful as an *invivo* model to study neurogenesis [16]. Ogita et al. [16] demonstrated that the administration of TMT induced acute neuronal death in the DG selectively 2 days later and that 5-bromo-2′-deoxyuridine (BrdU) incorporation into the DG cells was facilitated at an early stage (days 2–5) after the damage by TMT treatment. The BrdU-positive cells were also positive for nestin, NeuroD3, doublecortin or NeuN, suggesting that they were neural progenitor cells or neuronal precursors. From these results, they concluded that the hippocampal DG itself is capable of regenerating the neuronal cell layer through rapid enhancement of neurogenesis after TMT-induced injury.

Therefore, we investigated the effect of RJ on the neurogenesis of the DG using this animal model. We found that the oral administration of RJ for 6 days could significantly increase the number of Nissl-stained cells in the TMT-damaged DG on experimental day 8 when the TMT-induced neurogenesis would have ceased (Figure 3). It is conceivable that these generated neurons were functional, because RJ simultaneously ameliorated the cognitive impairment (Figure 2).

Our recent results highlighted a novel property of RJ, that is, that it facilitates the differentiation of all types of brain cells including neurons from cultured NS/NPCs [10], suggesting that RJ contains plural components that differently influence neuronal and/or glial lineages. Therefore, it might be expected that RJ or its components would facilitate *invivo* neurogenesis in the hippocampal DG. In fact, adenosine monophosphate (AMP) N_1_-oxide, which we earlier we identified to be a neurotrophic component of RJ [8], facilitates the generation of astrocytes from NS/PCs through activation of STAT3 [25]. On the other hand, 10-hydroxy-trans-2-decenoic acid, an unsaturated fatty acid characteristic of RJ, increases the generation of neurons and decreases that of astrocytes and oligodendrocytes from NSCs [10], suggesting that this compound unique to RJ is a predominant contributor to the neurogenesis.

Based on the results of the present study, we propose that RJ can facilitate neurogenesis *invivo*, which suggests that RJ may serve a tool for protection against and therapy for some particular neurological disorders such as depression, whose etiology is associated with reduced hippocampal DG neurogenesis [26]. RJ has the potential of being a promising evidence-based complementary and alternative medicine.
